# Lipid Metabolism in Oncology: Why It Matters, How to Research, and How to Treat

**DOI:** 10.3390/cancers13030474

**Published:** 2021-01-26

**Authors:** Yuki Matsushita, Hayato Nakagawa, Kazuhiko Koike

**Affiliations:** Department of Gastroenterology, The University of Tokyo, 7-3-1 Hongo, Bunkyo-ku, Tokyo 113-8655, Japan; matsushitay-int@h.u-tokyo.ac.jp (Y.M.); kkoike-tky@umin.ac.jp (K.K.)

**Keywords:** metabolic reprogramming, fatty acid metabolism, cholesterol metabolism, lipid droplet metabolism, phospholipid metabolism, tumor microenvironment, lipidomics, mass spectrometry

## Abstract

**Simple Summary:**

Metabolic reprogramming is gaining attentions as a hallmark of cancers. However, lipid metabolism has been difficult to analyze due to technical problems. In recent years, lipidomics techniques such as mass spectrometry have advanced and allowed us to analyze detailed lipid profiles of cancers. Moreover, it has become clear that changes in lipid metabolism also play an important role in the interaction between the cancers and the surrounding microenvironment. This review summarizes the latest research progress of reprogrammed lipid metabolism and also lipidomics technologies applied in cancer research.

**Abstract:**

Lipids in our body, which are mainly composed of fatty acids, triacylglycerides, sphingolipids, phospholipids, and cholesterol, play important roles at the cellular level. In addition to being energy sources and structural components of biological membranes, several types of lipids serve as signaling molecules or secondary messengers. Metabolic reprogramming has been recognized as a hallmark of cancer, but changes in lipid metabolism in cancer have received less attention compared to glucose or glutamine metabolism. However, recent innovations in mass spectrometry- and chromatography-based lipidomics technologies have increased our understanding of the role of lipids in cancer. Changes in lipid metabolism, so-called “lipid metabolic reprogramming”, can affect cellular functions including the cell cycle, proliferation, growth, and differentiation, leading to carcinogenesis. Moreover, interactions between cancer cells and adjacent immune cells through altered lipid metabolism are known to support tumor growth and progression. Characterization of cancer-specific lipid metabolism can be used to identify novel metabolic targets for cancer treatment, and indeed, several clinical trials are currently underway. Thus, we discuss the latest findings on the roles of lipid metabolism in cancer biology and introduce current advances in lipidomics technologies, focusing on their applications in cancer research.

## 1. Introduction

Lipids consist of numerous water-insoluble molecules and are mainly classified as fatty acids, triacylglycerides, phospholipids, or cholesterol. Lipids are widely distributed in cellular organelles and serve as building blocks of all the membranes. In addition to being components of biological membranes, lipids play an important role as energy sources, signaling molecules, and second messengers. Changes in metabolism have been considered a major characteristic of cancers. The most well-studied metabolic change is modification of glycolysis, the so-called Warburg effect [1]. Although research on the modification of cancer metabolism has increased since this discovery, it has mainly focused on the glycolytic system and glutamine metabolism. On the other hand, research on lipids, which are essential for living organisms, has been limited because they are water-insoluble; chemically unstable; and, unlike proteins, are not encoded by genes and must be analyzed directly using chemical methods. However, recent technological advances, such as mass spectrometry (MS), have fostered research into lipidomics. Lipidomics is the study of the structure and function of the complete set of lipids (the lipidome) produced in a given cell or organism, as well as their interaction with other lipids, proteins, and metabolites. Due to the wide range of polarities and the presence of structural analogs and isomers, lipid analysis requires advanced separation techniques. For the separation of lipids, not only the mass spectrometer alone but also the combination of various chromatography techniques have been used in different ways, depending on the target substance. Furthermore, new techniques known as mass spectrometry imaging (MSI) can be used for lipid localization to expand lipidomics further. The field of lipidomics, pioneered by these new technologies, is increasing our understanding of lipid metabolism reprogramming in cancers, and new therapeutic targets focusing on lipid metabolism have been identified. Therefore, in this review, we discuss the characteristic lipid metabolic changes in cancers and their pathological implications, as well as lipidomic techniques that have been applied.

## 2. Cell-Intrinsic Effects of Lipid Metabolic Reprogramming in Cancer Progression

### 2.1. Fatty Acids

#### 2.1.1. Basics of Fatty Acid Metabolism

Fatty acids (FAs) are important as the basic backbone of many lipids. The de novo FA synthesis pathway converts citrate to palmitic acid through sequential enzymatic reactions catalyzed by ATP citrate lyase (ACLY), acetyl-CoA carboxylase (ACC), and fatty acid synthase (FASN). Palmitic acid can be elongated by elongases (ELOVLs). These FAs can also be saturated and converted to monounsaturated fatty acids (MUFAs) by stearoyl-CoA desaturase (SCD) [2,3,4,5]. In addition to de novo synthesis, cells acquire FAs through uptake from extracellular sources. Lipid uptake is conducted through membrane-associated transport proteins, including fatty acid transport protein-1 (FATP1), fatty acid translocase (CD36), and liver fatty acid-binding protein (L-FABP). The expression of these enzymes related to FA metabolism are mainly regulated by sterol regulatory element-binding protein 1 (SREBP-1), known as the master transcription factor of lipogenesis [6].

**Figure 1:**
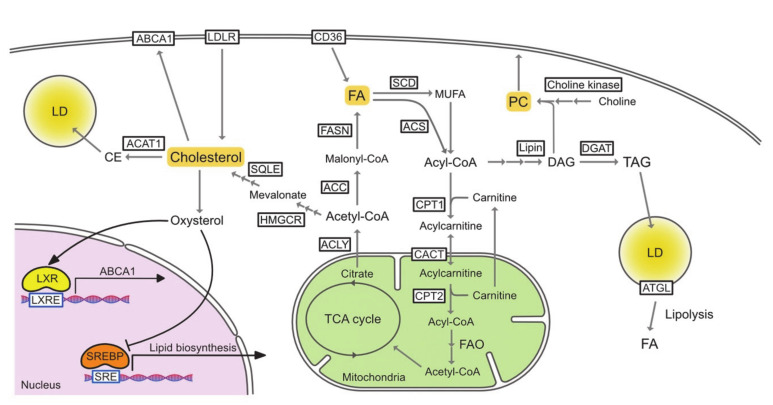
The pathways of lipid metabolism. ABCA1, ATP-binding cassette transporter A1; ACAT1, acetyl-CoA acetyltransferase 1; ACC, acetyl-CoA carboxylase; Acetyl-CoA, acetyl-coenzyme A; ACLY, ATP citrate lyase; ACS, acyl-CoA synthetase; Acyl-CoA, acyl-coenzyme A; ATGL, adipose triglyceride lipase; CACT, carnitine acylcarnitine translocase; CE, cholesterol ester; CPT1, carnitine palmitoyltransferase 1; CPT2, carnitine palmitoyltransferase 2; DAG, diasylglycerol; DGAT, diglyceride acyltransferase; FA, fatty acid; FAO, fatty acid oxidation; FASN, fatty acid synthase; HMGCR, 3-hydroxy-3-methylglutaryl-CoA reductase; LD, lipid droplet; LDLR, low-density lipoprotein receptor; LXR, liver X receptor; LXRE, LXR response element; MUFA, monounsaturated fatty acid; PC, phosphatidylcholine; SCD, stearoly-CoA desaturase; SRE, sterol regulatory element; SREBP, sterol regulatory element-binding protein; SQLE, squalene epoxidase; TAG, triacylglycerol; TCA, tricarboxylic acid.

To be used in metabolic pathways, FAs must be activated by acyl-CoA synthetase (ACSLs), which converts free FAs to acyl-CoA. In the fatty acid oxidation (FAO) process, the rate-limiting step is the translocation of acyl-CoA across the mitochondrial membrane. Through this translocation, acyl-CoA is first converted to acylcarnitine via its conjugation to carnitine by carnitine palmitoyltransferase 1 (CPT1). Acylcarnitine is then translocated into the mitochondria via carnitine acylcarnitine translocase (CACT) and finally converted back to acyl-CoA by CPT2. Acyl-CoA then enters the FAO pathway and is followed by the tricarboxylic acid (TCA) cycle (Figure 1).

#### 2.1.2. Reprogrammed Fatty Acid Metabolism in Cancer Cells

De novo fatty acid synthesis is activated in several cancers and has been extensively reviewed elsewhere [2,7,8,9]. Therefore, we briefly mention the change in de novo FA synthesis in cancer cells. Enzymes that catalyse de novo FA synthesis including ACLY, ACC, FASN, and SCD are upregulated in numerous cancers [9,10,11,12,13,14]. Several chemical inhibitors targeting these enzymes are currently in preclinical and clinical trials for cancer treatment. We summarize these inhibitors in Table 1.

In addition to de novo synthesis, interrupting FA uptake is known to be effective in cancer therapy. CD36 expression is upregulated in ovarian cancer, gastric cancer, glioblastoma, and oral squamous cell carcinoma [43]. The anti-tumor effect of SCD inhibitors can be reversed by exogenous oleic acid, but in CD36 knockdown breast cancer cells, the effect cannot be reversed [37]. Thus, combination therapy targeting lipogenesis and lipid uptake would be a promising approach. Two CD36-targeting drugs, ABT-510 and CVX-045, reached clinical trials but eventually both failed due to severe adverse effects and unsatisfactory efficacy [44]. Preclinical studies with other agents are currently underway [43].

Cancer cells frequently show changes in enzymes involved in FAO. FAO is an important bioenergetic pathway in many cancers that promotes proliferation, metastasis, stemness, and resistance to treatment [45,46]. Overexpression of CPT1 is associated with tumor progression in several types of cancers such as breast cancer, gastric cancer, and prostate cancer [47]. CPT1 inhibitors such as etomoxir and phexiline were expected to have antitumor effects but have not progressed to clinical studies due to their strong side effects [47]. Nevertheless, a more selective CPT1 inhibitor named ST1326 shows cytotoxic effects against acute myelogenous leukemia (AML) cells and is expected to be clinically applied [48]. On the other hand, suppression of FAO is sometimes beneficial for cancer cell growth. We previously reported that, in patients with nonalcoholic steatohepatitis (NASH), CPT2 downregulation-mediated suppression of FAO not only enables hepatocellular carcinoma (HCC) cells to escape lipotoxicity, but also promotes hepatocarcinogenesis through the accumulation of acylcarnitine as an oncometabolite [49]. Decreased expression of CPT2 in HCC have been confirmed by other recent studies [50,51]. In colon cancer, CPT2 expression is negatively correlated with tumor stage and low expression of CPT2 is associated with poor prognosis [52]. Glioblastoma cells store excess FAs into triglycerides and lipid droplets (LDs) by upregulating diacylglycerol-acyltransferase 1 (DGAT1), and inhibition of DGAT1 promotes glioblastoma cell death through excessive FAO-mediated reactive oxidative species (ROS) production [53]. These findings suggest that the role of FAO in carcinogenesis varies depending on cancer type and the surrounding microenvironment. Of note, we found that an altered serum acylcarnitine profile caused by downregulation of CPT2 is a useful marker for predicting HCC in patients with NASH [54]. Therefore, specific metabolites generated by the altered lipid metabolism may be biomarkers for cancer.

### 2.2. Cholesterol

#### 2.2.1. Basics of Cholesterol Metabolism

Cholesterol is present in all cell membranes and plays an important role in the regulation of membrane function. The cholesterol content can control the fluidity and flexibility of the membrane. In addition, when present with sphingolipids, the two form clusters known as lipid rafts, regulating the two-dimensional distribution of membrane proteins. Typically, signal transduction-related proteins are believed to rely on these rafts. In mammalian cells, cholesterol is synthesized from acetyl-CoA through the mevalonate pathway. First, hydroxymethylglutaryl-CoA (HMG-CoA) is synthesized from three acetyl-CoA molecules. HMG-CoA is then reduced to mevalonate by HMG-CoA reductase (HMGCR). In a series of enzymatic reactions, mevalonate is converted to farnesyl pyrophosphate (FPP). The two FPP molecules are condensed to squalene and then oxidized by squalene epoxidase (SQLE) to 2,3-epoxy squalene, which is cyclized to lanosterol. Lanosterol is eventually converted to cholesterol [55]. 

Besides cholesterol biosynthesis, most cells acquire extracellular cholesterol from low-density lipoprotein (LDL) via the LDL receptor (LDLR) [56]. On the other hand, excess cholesterol is exported from cells by ATP-binding cassette (ABC) transporters, including ABCA1 and ABCGs, or converted to less toxic cholesteryl esters (CEs) by acyl CoA:cholesterol acyltransferases (ACATs). These CEs are stored in LDs or secreted into lipoproteins [57]. 

Cholesterol concentrations are tightly controlled by SREBP-2, liver X receptor (LXR), and nuclear factor erythroid 2-related factor-1 (NRF1). Accumulation of cholesterol and cholesterol-derived oxysterols inactivate the SREBP-2 pathway by inducing the retention of the SREBP cleavage-activating protein (SCAP)–SREBP-2 complex in the endoplasmic reticulum (ER) via the insulin-inducing gene (INSIG), which downregulates the biosynthesis and uptake of cholesterol [58]. On the other hand, desmosterol and oxysterol bind and activate LXRs, which enhances the expression of genes involved in cholesterol efflux [59]. High cholesterol levels inhibit nuclear translocation of NRF1 and restore the LXR pathway that is blocked by NRF1 [60]. In cholesterol deficiency, the three regulatory pathways work in a coordinated manner to increase cholesterol biosynthesis and uptake, while decreasing cholesterol efflux and esterification (Figure 1).

#### 2.2.2. Reprogrammed Cholesterol Metabolism in Cancer Cells

**Figure 2:**
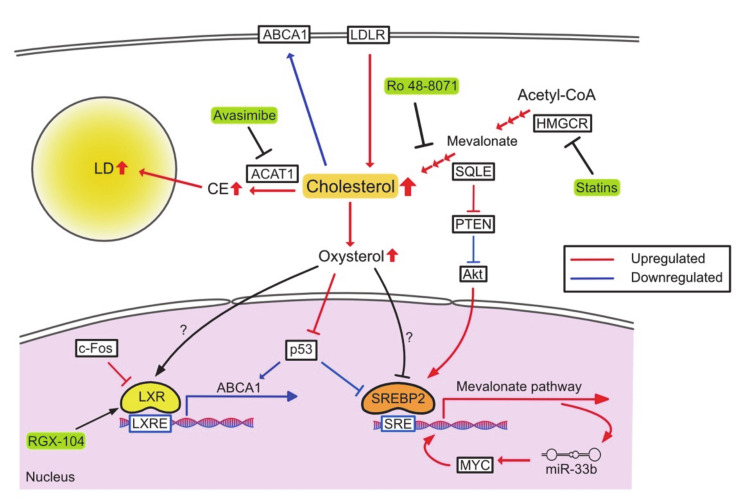
Reprogrammed cholesterol metabolism and therapeutic targets. PTEN, phosphatase and tensin homolog deleted from chromosome 10.

Because of their rapid proliferation, cancer cells require high levels of cholesterol, and increased cholesterol biosynthesis is a hallmark of many cancers (Figure 2). SREBP2 and its target genes are markedly upregulated in prostate cancer, breast cancer, and HCC [61]. Cholesterol biosynthesis also plays an important role in the maintenance of cancer stem cells by activating sonic hedgehog and Notch pathway [62]. 

Since de novo cholesterol synthesis is time- and energy-consuming, some cancers such as glioblastoma and pancreatic cancer utilize exogenous cholesterol [63,64]. Some anaplastic large cell lymphomas lack SQLE and aggressively upregulate LDLR to acquire cholesterol [65]. Furthermore, loss of SQLE leads to the upstream accumulation of squalene, which protects cells from ferroptosis by maintaining an appropriate composition of membranous polyunsaturated fatty acids (PUFAs). 

Accumulation of CE is another characteristic of cancer. ACAT1 plays a tumor-promoting role in pancreatic cancer and lymphocytic leukemia [66,67]. Oxysterols are other cholesterol metabolites found abundantly in the tumor microenvironment (TME) [68]. In estrogen receptor-positive breast cancer patients, 27-hydroxycholesterol (27HC) is accumulated in both breast tissue and tumor [69]. 27HC promotes cell proliferation by enhancing the function of p53 E3 ligase MDM2 and inhibiting p53 activation in breast cancer cells [70]. On the other hand, oxidized sterols are known to be LXR ligands and inhibit cell proliferation [71]. 27HC, 24(R/S), and 25-epoxycholesterol inhibit growth and metastasis of gastric cancer by activating LXR signaling [72]. Thus, the roles of 27HC in cancers remain contradictory [73].

The acquisition of oncogenes and the loss of tumor suppressor genes are associated with changes in cholesterol metabolism. The oncogene MYC is required for upregulation of the mevalonate pathway in brain tumor cells, and the upregulation of this pathway forms a positive feedback that increases the expression of the microRNA miR-33b, which in turn increases expression of MYC [74]. In hepatocytes, transgenic expression of c-Fos, an oncogene, suppresses LXR signaling and increases the synthesis of cholesterol and cholesterol metabolites such as oxysterol and bile acids. This leads to increased inflammation and hepatocarcinogenesis [75]. On the other hand, p53 suppresses the mevalonate pathway by upregulating the expression of ABCA1 and limiting the activation of SREBP2 [76]. In addition to de novo cholesterol synthesis, tumor suppressor genes also restrict cholesterol uptake and esterification. In prostate cancer, loss of phosphatase and tensin homolog (PTEN) activates the PI3K-Akt pathway and increases cholesterol uptake and esterification, leading to CE accumulation [77]. SQLE expression is upregulated in NASH-derived HCC, which suppresses PTEN expression and activates Akt signaling, thereby increasing CE and inducing carcinogenesis [78]. 

Inhibition of cholesterol metabolism has been considered a feasible anti-tumor therapy [79,80]. Statins, well-known HMGCR inhibitors, reduce mortality in various cancer types, regardless of use before or after diagnosis [81,82,83,84], and have also been studied as anti-tumor drugs in clinical trials [85,86]. Another cholesterol synthetic enzyme, SQLE, is considered to be a target for anti-tumor therapy [87]. Several drugs for SQLE have been certified as antifungal agents and are being investigated for use as antitumor agents [55]. Ro 48-8071 (oxidosqualene cyclase inhibitor) significantly inhibits growth and metastasis of colorectal and pancreatic cancer [88]. Co-therapy of this inhibitor with 5-fluorouracil (5-FU) also shows a higher antitumor effect. Targeting cholesterol uptake with LXR agonists could cause LDLR degradation and increase the expression of ABCA1, promoting tumor cell death in numerous cancers [89,90,91]. Inhibition of cholesterol esterification is also an effective approach. CE inhibition by the ACAT1 inhibitor avasimibe suppresses tumor growth and restores imatinib sensitivity by downregulating MAPK signaling in imatinib-resistant myeloid leukemia [92]. In prostate cancer, avasimibe inhibits metastasis by blocking the Wnt-β-catenin pathway [93]. Avasimin (human albumin-capsulated avasimibe) specifically induces tumor apoptosis in a tumor xenograft model [94].

### 2.3. Triacylglycerol/Lipid Droplet

#### 2.3.1. Basics of Triacylglycerol/Lipid Droplet Metabolism

Triacylglycerol (TAG) is composed of three fatty acids esterified to a glycerol molecule. The basic role of this molecule is storing fatty acids and promoting efficient transport. The largest source of TAG is the diet, and each cell takes up TAG as FAs. In adipocytes and hepatocytes, these FAs can be resynthesized into TAG. Acyl-CoA derived from FA is converted to TAG through a series of reactions catalyzed by glycerol-3-phosphate acyltransferase (GPAT), acylglycerol phosphate acyltransferase (AGPAT), phosphatidic acid phosphohydrolase (Lipin), and diacylglycerol acyltransferase (DGAT) [3]. TAGs are then sequestered within LDs. 

LD is an organelle that has a neutral lipid core that is surrounded by a phospholipid monolayer. The internal lipid is mainly composed of TAG, but also contains CEs and acyl ceramides. The formation of LDs begins with de novo synthesis of TAGs and CEs between the lipid bilayers of the ER [95]. When sufficient lipids have accumulated in the ER intermembrane lens, they are released into the cytoplasm to form LDs. LDs are dynamically synthesized and degraded in response to the extracellular nutrient environment. LDs are mainly degraded by two mechanisms, namely, lipolysis and lipophagy [96,97]. In lipolysis, FAs can be supplied from TAGs in reactions with adipose triglyceride lipase (ATGL), hormone-sensitive lipase (HSL), and monoacylglycerol lipase (MAGL) [98,99] (Figure 1). Lipophagy is a recently discovered form of autophagy in which LDs are incorporated into autophagosomal membranes, fused with lysosomes, and hydrolyzed [100]. FAs supplied by the degradation of LDs fuel the mitochondrial oxidative metabolism during nutrient-deprived conditions in various cells [101]. On the other hand, lipolysis may lead to lipotoxicity. Excess lipolysis increases harmful free FAs in the cytoplasm and also increases FA oxidation in mitochondria, leading to the production of ROS [102]. Perilipin 5 inhibits ATGL-mediated lipolysis and protects tissues against these forms of oxidative stress [103].

#### 2.3.2. Reprogrammed Triacylglycerol/Lipid Droplet Metabolism in Cancer Cells

During carcinogenesis, the demand for FA increases to support rapid proliferation. FA is supplied not only by increased de novo synthesis but also by increased uptake from extracellular sources and recycling of intracellular lipids through autophagy, which requires contribution of lipid droplets. The lipid droplet also plays an important role in defending against ROS and developing resistance to stress from the harsh surrounding environment, such as hypoxia and low nutrition [101,104,105]. Lipid droplets are known to accumulate in various types of cancer [106], and tumors with high concentrations of lipid droplets are associated with a poor prognosis in breast and pancreatic cancer [64,107]. Thus, changes in lipid droplet metabolism are also an important hallmark of cancer. 

Hypoxia-associated lipid droplet production has been observed in brain, breast, renal, and prostate cancers [105,108,109,110,111,112,113]. HCC and cervical adenocarcinoma cells exposed to hypoxia accumulate lipid droplets by directly stimulating the expression of Lipin 1 in a HIF-1α-dependent manner [110]. Cancer cells exposed to hypoxia are known to upregulate FA uptake via FABP3/7 and accumulate lipid droplets, enabling ER and redox homeostasis during oxygen deprivation, supplying FA for mitochondrial energy production and promoting cell proliferation after reoxygenation [105]. 

Recent studies have shown that hypoxia can affect the expression of several lipid droplet-related proteins involved in lipid droplet degradation [114,115]. Hypoxia-inducible lipid droplet-associated protein (HILPDA)/hypoxia-inducible gene 2 (HIG2), localized on the lipid droplet surface, is an inhibitor of ATGLs, which are upregulated by HIF-1 during hypoxia [114,115,116]. HIG2 deficiency promoted lipid droplet degradation and β-oxidation, resulting in elevated ROS production and impaired xenograft tumor growth. Conversely, ATGL knockout (KO) reversed the effect of HIG2 KO, and ATGL inhibition by HIG2 inhibited lipid droplet degradation and isolated FA from mitochondrial oxidation and ROS production, allowing for cancer survival under hypoxic conditions [114]. Thus, lipid droplet degradation by ATGL may damage cancer cells under hypoxic conditions and increasing ATGL activity may be a novel therapeutic strategy. Indeed, ATGL overexpression represses proliferation of melanoma, colon carcinoma, HCC, and lung cancer cell lines [117,118], and also enhances the response of anti-cancer agents against HCC cell lines [118].

The role of lipophagy in cancer remains unclear. Although several reports have shown a tumor-promoting effect of lipophagy [119,120], most of them state that lipophagy inhibits tumorigenesis [121,122,123,124]. Microtubule-associated protein 1S (MAP1S)-mediated lipophagy promotes the elimination of lipid droplets, and high expression of MAP1S is associated with the suppression of tumor growth and metastasis and an improved prognosis in clear-cell renal cell carcinoma [121]. Another study showed that overexpression of ATG14 promotes lipid droplet degradation in HeLa cells and induces free FA accumulation, leading to ER stress and ROS-mediated apoptosis, while inhibition of lipophagy by 3-methyladenine (3-MA) and inhibition of lysosomal acid lipase (LAL) reverses these effects [122]. Recent studies have suggested that LAL plays a role as a tumor suppressor, and that impaired LAL function in mice is associated with spontaneous tumorigenesis. Re-expression of LAL in the liver and lungs improves inflammation and prevents metastasis to the same areas [123,124]. 

### 2.4. Phospholipid

#### 2.4.1. Basics of Phospholipid Metabolism

Phospholipids (PLs) are components of the cell membrane and have diverse chemical structures and functions. PLs regulate various cellular functions such as homeostasis, cell adhesion and migration, signal transduction, vesicle transport, apoptosis, and post-translational modifications. Glycerophospholipids (GPLs) are classified into subclasses known as phosphatidylcholine (PC), phosphatidylethanolamine (PE), phosphatidylserine (PS), phosphatidylglycerol (PG), phosphatidylinositol (PI), and phosphatidic acid (PA), depending on the type of polar head group. The fatty acyl moieties of the membrane phospholipids show considerable diversity in chain length and saturation. These two parameters determine the biophysical properties of the cell membrane, such as fluidity, curvature, and subdomain structure. These factors affect membrane-related cellular processes, such as signal transduction, and molecular transport [125]. 

For de novo PL synthesis, FA is first taken up by phosphatidic acid (PA) as the main precursor of PL. The Kennedy pathway is the primary pathway for the synthesis of PC, which is the most abundant PL head group class [126]. The pathway contains three enzymatic reactions: choline phosphorylation by choline kinase, CDP-choline formation from phosphocholine and CTP catalyzed by CTP:phosphocholine cytidylyltransferase (CCT), and substitution of cytidine monophosphate by diasylglycerol (DAG) to produce PC catalyzed by CDP-choline:1,2-diacylglycerol cholinephosphotransferase (Figure 1). The next most common PL is PE, which can be synthesized de novo but can also be generated from PS by head group exchange. Besides de novo synthesis and head group exchange, the phospholipid composition is maintained through a deacylation and reacylation remodeling process called the Lands’ cycle [127]. Lysophosphatidylcholine acyltransferases (LPCATs) play an important role in lipid metabolism and homeostasis by regulating different PC species in multiple cells and tissue types. LPCAT1 is mainly expressed in alveolar type II cells and catalyzes the generation of dipalmitoyl-PC (DPPC) in lung surfactants [128]. LPCAT2 is highly expressed in inflammatory cells, such as macrophages and neutrophils, and is also present in the skin, colon, spleen, and brain [129]. In contrast, LPCAT3 is more widely expressed and abundant in the testes, kidneys, and metabolic tissues including liver, intestines, and fat. LPCAT4 is selectively expressed in the epididymis, brain, testes, and ovaries [130]. LPCAT1 and LPCAT2 are known to have lyso-platelet activating factor (PAF) acetyltransferase activity in PAF biosynthesis, in addition to LPCAT activity [129]. Most importantly, each LPCAT shows a different acyl-CoA preference. LPCAT1 prefers palmitoyl-CoA (16:0-acyl-CoA) as the acyl donor for synthesizing dipalmitoyl PC [128]. LPCAT2 shows the highest activity in the presence of acetyl-CoA or arachidonoyl CoA (20:4-acyl-CoA) [129]. In contrast, LPCAT3 and LPCAT4 prefer polyunsaturated fatty acyl-CoA (18:2-acyl-CoA or 20:4-acyl-CoA) and oleoyl-CoA (18:1-acyl-CoA) as substrates, respectively [130,131]. Thus, the different substrate preferences and tissue expression patterns of LPCAT contribute to tissue-selective remodeling of membrane PC species.

#### 2.4.2. Reprogrammed Phospholipid Metabolism in Cancer Cells

Many of the enzymes involved in PL synthesis and remodeling are overexpressed in cancer. For example, Lipin-1, which regulates the rate-limiting step in PL synthesis and is a co-regulator of transcription factors such as peroxisome proliferator-activated receptors (PPARs) and SREBPs, is upregulated in a diverse subset of cancer types, including high-grade prostate, colon, lung, and breast cancer [131,132,133]. High levels of Lipin-1 expression are associated with a poor prognosis and inflammation, and downregulation of Lipin-1 induces ER stress and apoptosis and attenuates tumor growth in xenograft mouse models. Choline kinase alpha (ChoKalpha), the first committed enzyme of the Kennedy pathway for PC and PE synthesis, is overexpressed in a variety of tumor types and is activated by various oncogenic events. Activation and overexpression of ChoKalpha is associated with increased cellular requirement for PC. Knockdown or chemical inhibition of ChoKα causes cell death and attenuates tumor growth in vivo [134,135].

Another emerging class of enzymes that appear to be affected in many cancers is LPCAT. Upregulation of LPCAT1 has been observed in clear renal cell carcinoma, oral squamous cell carcinoma, hepatoma, esophageal cancers, gastric cancers, breast cancers, colorectal cancers, and prostate cancers [136]. LPCAT1 expression is correlated with prognosis and survival in clear cell renal cell carcinoma, breast cancer, and prostate cancer [137,138,139,140] and can be used as a diagnostic marker in prostate cancer and esophageal cancer [139,141]. Overexpression of LPCAT1 has been shown to increase cell proliferation, migration, and metastasis in clear cell renal cell carcinoma and HCC cell lines [137,142]. Consistent with LPCAT1 enzyme activity, the level of saturated phospholipids is increased in clear cell renal cell carcinoma, HCC, and gastric cancers [137,142,143]. However, it remains unclear as to how these changes in PC levels regulate the behavior of cancer cells. LPCAT1 may also contribute to tumor growth through lyso-PAF acetyltransferase activity to produce PAF, a lipid mediator that plays an important role in cell growth [144,145]. LPCAT2 has also been reported to be overexpressed in cervical and breast cancers [146], and has been identified as a susceptibility gene for aggressive prostate cancer in animal models and genome-wide association studies in human patients [147]. LPCAT2-mediated LD production is known to contribute to chemotherapy resistance in colorectal cancer. Thus, targeting LPCAT2-mediated intracellular LD formation may be a therapeutic approach to restore chemotherapy sensitivity in colorectal cancer [148]. Loss of LPCAT3 in the mouse gut reduces the composition of polyunsaturated phospholipids and promotes tumor development and growth in Apc^min^ mice [149]. LPCAT4 is associated with intestinal stem cell proliferation and tumorigenesis, and is also associated with high levels of PC (16:0/16:1) in colorectal cancer [135].

## 3. Altered Lipid Metabolism and Tumor Microenvironment

**Figure 3:**
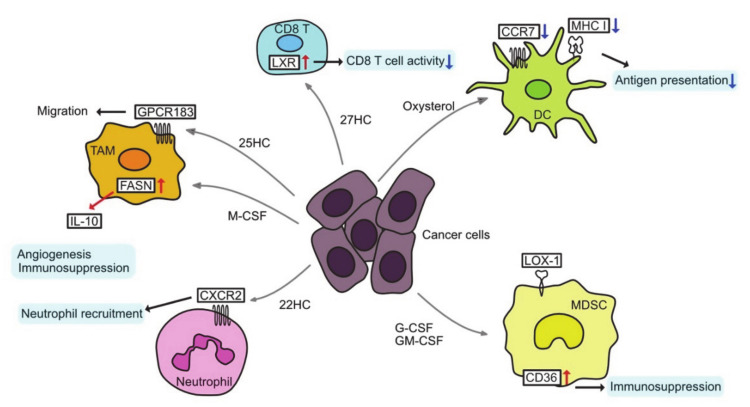
Altered lipid metabolism and tumor microenvironment. CCR7, C-C chemokine receptor type 7; CXCR2, C-X-C chemokine receptor type 2; DC, dendritic cell; G-CSF, granulocyte colony-stimulating factor; GM-CSF, granulocyte macrophage colony-stimulating factor; GPCR 183, G-protein-coupled receptor 183; HC, hydroxycholesterol; IL-10, interleukin-10; LOX-1, lectin-like oxidized low-density lipoprotein (LDL) receptor-1; M−CSF, macrophage colony stimulating factor; MDSC, myeloid-derived suppressor cell; MHC I, major histocompatibility complex class I; TAM, tumor-associated macrophage.

In addition to cancer cells, tumors contain a variety of immune-effector and immunosuppressive cells, termed tumor-infiltrating immune cells (TIIs). TIIs range from anti-tumor to tumor-promoting functions, and they depend on the type and stage of the tumor [150]. TIIs include T lymphocytes, B lymphocytes, tumor-associated macrophages (TAMs), dendritic cells (DCs), myeloid derived suppressor cells (MDSCs), neutrophils, and natural killer (NK) cells (Figure 3).

### 3.1. Tumor-Associated Macrophages

TAMs can be reprogrammed as a result of changes in cholesterol metabolism. Tumor cells secrete hyaluronan oligomers that increase cholesterol efflux in the TAMs, leading the TAMs to the M2-like phenotype and accelerating tumor progression [151]. 25-Hydroxycholesterol (25HC) interacts with G-protein-coupled receptor 183 (GPCR183) to reconstitute the cytoskeletal protein vimentin, resulting in the migration of macrophages and monocytes [152]. Moreover, in the context of changes in FA metabolism, TAMs can be polarized to a pro-tumoral phenotype. Macrophage colony-stimulating factor (M-CSF) secreted from tumor cells upregulates FASN expression in TAMs, leading to PPARβ/δ activation and IL-10 expression, which is an anti-inflammatory cytokine [153].

### 3.2. T cells in TME

In CD8 T cells, SREBP2 signaling is essential for proliferation and effector function, whereas LXR signaling negatively regulates T cell activation [154,155]. Therefore, in oxysterol-rich TMEs, T cell tumor immunity may be inhibited by LXR activation. On the other hand, increased cholesterol synthesis and uptake by T cells may enhance the antitumor effect of T cells. Inhibition of ACAT1 in CD8 T cells alters cholesterol synthesis and leads to an accumulation of free cholesterol in the plasma membrane [156]. This cholesterol binds directly to T cell receptors and promotes nanoclustering, which triggers antigen-induced signals that increase cholesterol biosynthesis and uptake [157]. Furthermore, this cholesterol plays a role in the formation of mature immunological synapses for targeted killing of tumor cells. The ACAT1 inhibitor, avasimibe, enhances the therapeutic effect of chimeric antigen receptor (CAR)-T cells by increasing the ratio of cytotoxic CD8 T cells [158]. On the other hand, cholesterol accumulation in TME has been shown to induce ER stress and further increase T cell exhaustion [159]. Thus, the functions of endogenous and exogenous cholesterol may differ.

### 3.3. Tumor-Associated Dendritic Cells

Conditioned medium of multiple cancer cells activates LXR-alpha signaling in dendritic cells and reduces cell surface expression of CC chemokine receptor-7 (CCR7) [160]. As a result, the migration of dendritic cells from the tumor site to the lymph nodes is inhibited and the presentation of tumor antigens to T cells is suppressed. Inactivation of LXR-α ligand through the inhibition of cholesterol synthesis or expression of SULT2B1b (an enzyme that converts oxysterol to inactive sulfated oxysterol) in tumor-bearing mice restores dendritic cell function and antitumor response [160]. However, it remains unclear as to which oxysterol is responsible for this effect. Tumor-derived factors result in the accumulation of oxidized lipids such as CE, TAG, and FA, which reduce major histocompatibility complex (MHC) class I complexes on the surface of dendritic cells and decrease antigen presentation [161]. LD accumulation in DCs also inhibits their antigen-presenting ability in kidney, thyroid, ovarian, and head and neck cancer [162]. LPCAT-2-dependent lipid droplet accumulation in colorectal cancer causes calreticulin sequestration and prevents its exposure to the plasma membrane, thereby preventing DC maturation and subsequent CD8 T cell infiltration and immunogenic cell death under chemotherapy [148].

### 3.4. Immunosuppressive Cells

Neutrophils are considered an important immunosuppressive population in TME [163,164]. 22HC is abundant in the conditioned medium of various cancer cells and can mobilize CD11bhighGr1high neutrophils [165]. 22HC mobilizes neutrophils by binding to CXCR2, but not LXR. 24HC and 27HC can also mobilize neutrophils in other carcinomas. Pancreatic neuroendocrine tumors show that HIF1α-induced increases in 24S-HC levels induce neutrophils and angiogenesis [166]. 27HC promotes metastasis by attracting polymorphonuclear neutrophils and γδ T cells and reducing cytotoxic CD8 T cells in a high-cholesterol diet-fed breast cancer model [167].

MDSCs are very similar to neutrophils and are considered as immunosuppressive innate cell populations. They have unique characteristics, and LOX-1 (lectin-type oxidized LDL receptor-1) has recently been characterized as an important marker to distinguish them from neutrophils [168]. Overexpression of LOX-1 has been found in several cancers and is associated with a poor prognosis. LOX-1 is an LDL receptor, suggestive of altered cholesterol metabolism in MDSCs. LXR agonist RGX-104 depletes MDSCs and enhances T cell activity via upregulation of APOE [169]. LXR activation enhances the efficacy of immunotherapies such as immune checkpoint inhibitors and adoptive T cell transplantation [170,171]. In addition, granulocyte colony-stimulating factor (G-CSF) and granulocyte macrophage colony-stimulating factor (GM-CSF) secreted from cancer cells act via signal transducer and activator of transcription (STAT)3/5 in a paracrine manner on MDSCs, resulting in overexpression of CD36 and enhancement in uptake of FAs. Thus, STAT3 or STAT5 inhibition, or CD36 deletion, are known to downregulate lipid metabolism and prevent the immunosuppressive functions of MDSCs [172].

## 4. Lipidomic Research Techniques

**Figure 4:**
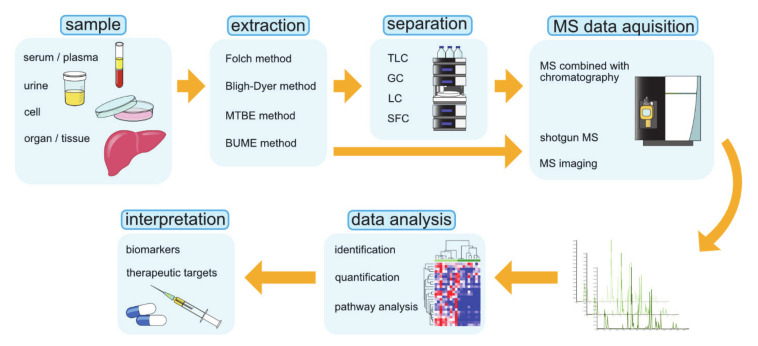
The operating procedures in lipidomics research. BUME, butanol–methanol; GC, gas chromatography; LC, liquid chromatography; MS, mass spectrometry; MTBE, methyl-tert-butyl-ether; SFC, supercritical fluid chromatography; TLC, thin-layer chromatography.

Lipidomics is the large-scale profiling and quantification of lipid molecules, a study that comprehensively examines lipid pathways and interprets their physiological significance on the basis of analytical chemistry and statistical analysis [173,174]. Lipidomic research can be used to accumulate a vast amount of information that quantitatively describes spatial and temporal changes in the content and composition of lipid molecular species after perturbations such as diseases, drugs, and the environment. The goal of lipidomics is to explore the basic mechanisms of lipid metabolism and its changes under pathological conditions, which may reveal biomarkers that can be used to diagnose and treat diseases, target drug discovery, guide precision and personalized medicine, and intervene in dietary habits [175]. The commonly used operating procedures of lipidomics are graphically summarized in Figure 4 [175].

The advances in techniques such as MS, NMR spectroscopy, and chromatography have contributed greatly to the development of lipidomics. Above all, MS is the fundamental technology that is widely used in lipidomics. MS-based lipidomics can be divided into two subfields, namely, non-targeting lipidomics and targeted lipidomics. Non-targeted lipidomics, or so-called global lipidomics, identifies and quantifies all detected lipids, whereas targeted lipidomics is focused on the analysis of the specific lipid class. While non-targeted lipidomics is a suitable method for capturing the rough profile of lipids in the sample, targeted lipidomics may be more applicable to solving specific biological problems. In lipidomics, three MS methods are mainly used: direct injection shotgun MS, MS combined with chromatographic separations, and mass spectrometric imaging (MSI) [175]. A summary of these techniques is given in Table 2.

### 4.1. Shotgun MS

Shotgun MS is an analytical method in which lipid extracts are injected directly into the MS without prior chromatographic separation. It is a less time-consuming, more convenient, and more reproducible method compared to other methods. However, shotgun lipidomics is limited for detecting lipid molecules at relatively low concentrations because ionization is suppressed due to the complex matrix, and in many cases, they could not be detected. In addition, with shotgun lipidomics, lipid isomers are not distinguished by shotgun method. To address these problems, collaboration between chromatography equipment and MS is essential.

### 4.2. MS Coupled with Chromatography

Chromatographic methods are important for reducing matrix effects, separating lipid isomers, and condensing lipid molecules. These techniques are necessary for analysis of a wide variety of lipids from biological samples. Separation techniques include thin-layer chromatography (TLC), gas chromatography (GC), liquid chromatography (LC), and supercritical fluid chromatography (SFC).

#### 4.2.1. TLC: Thin-Layer Chromatography

In TLC, a thin film of adsorbent such as silica gel on a glass or aluminum plate is used as the stationary phase, and liquid is used as the mobile phase. Sample materials can be separated by the variation in the transfer distance due to the difference in the strength of attachment to the adsorbent and the solubility to the solvent. TLC can separate lipid mixtures into individual lipid categories more quickly and inexpensively than LC or GC. However, the most problematic aspect of TLC analysis is separation efficiency and sensitivity. Low separation efficiency and sensitivity results in poor resolution and requires more sample. Therefore, it is not suitable for the separation of small amounts of biological samples. Furthermore, due to its structure, it is not possible to link TLC and MS detection directly to improve sensitivity. TLC and matrix assisted laser desorption/ionization (MALDI)–MS can, typically, be combined offline [186,187].

#### 4.2.2. GC: Gas Chromatography

GC is a separation technique that uses a gas as the mobile phase and is used for the analysis of volatile components. He, N_2_, H_2_, etc. are used as the carrier gas, which is the mobile phase. The sample is heated and vaporized, and is introduced into the column by the carrier gas. The sample is separated by the difference in volatility and the interaction with the stationary phase, coated on the inner surface of the column. Therefore, GC is suitable for the separation of volatile compounds that are resistant to heat, and it has a high separation efficiency. For compounds that are difficult to volatilize, the derivatization process can be used for analysis. Derivatization is also used to improve the separation. This combined GC and MS method is currently the dominant method for profiling and quantification of FA and cholesterol [188,189,190]. Its relative low cost is another advantage. On the other hand, there is the disadvantage in that non-volatile substances and substances with low heat stability require prior derivatization processing.

#### 4.2.3. LC: Liquid Chromatography

LC, with its high sensitivity and ease of connection with MS, is the most widely used chromatographic method. The separation modes of LC can be broadly divided into normal-phase and reversed-phase systems. In general, normal-phase separates lipids on the basis of the headgroup of the lipid compound. On the other hand, reversed-phase separates lipids according to the hydrophobicity, length, and number of double bonds of the FA chain. Thus, the separation mode should be chosen on the basis of where in the lipid structure there will be a focus. High-performance liquid chromatography (HPLC) is commonly used in lipidomics due to its high separation power and selectivity. In recent years, ultra high performance liquid chromatography (UHPLC) has been developed and rapidly spread. The use of high-pressure resistant columns and smaller size particles gives UHPLC higher separation efficiencies compared to conventional HPLC. UHPLC–MS enables high sensitivity, high resolution, and high speed of lipidomic analysis. The limitations of LC–MS are consumption of organic solvent, which is not environmentally friendly, requires pre-knowledge for targeted analysis, and involves complex data processing.

#### 4.2.4. SFC: Supercritical Fluid Chromatography

SFC is a high-resolution, high-throughput separation method that uses supercritical fluid (a non-condensable fluid that exceeds the inherent gas–liquid critical point of a substance) as the mobile phase. It has the properties of both GC and HPLC; in addition, it is possible to select a wide range of separation modes by changing the state of the mobile phase. Although supercritical carbon dioxide, which is the most commonly used solvent, is low polarity, the polarity of the mobile phase can be changed by adding a polar organic solvent including methanol as a modifier. Therefore, a wide range of substances, from polar to nonpolar, can be separated. The disadvantage of this technology is that it is new, and thus there is little information about reproducibility, and it is not easily accessible.

### 4.3. MS Imaging

MSI is a relatively new MS-based imaging technique that uses direct ionization and MS detection to provide visualization and distribution of individual lipid molecules in tissue samples. MALDI, secondary ion mass spectrometry (SIMS), and desorption electrospray ionization (DESI) are the three ionization techniques used in MSI [191]. MALDI–MSI is the most commonly used technique, in which a matrix is applied to the sample surface to assist in the ionization. A wide range of molecules including lipids can be observed without fragmentation and with a spatial resolution of up to several micrometers. SIMS is a technique to obtain secondary ions of sample molecules by irradiating ionized noble gas or metal atoms. SIMS requires no pretreatment and has high spatial resolution on the submicron scale. Because it is a hard ionization method, fragmentation of lipid molecules is likely to occur, making it suitable for analyzing differences in fatty acid composition, headgroups, and other components of lipid molecules with high spatial resolution. DESI extracts, desorbs, and ionizes molecules by spraying charged microdroplets on the sample. It is a soft ionization technique that does not require matrix pretreatment, unlike MALDI, and can detect free fatty acids and lipid mediators, which are difficult to detect with MALDI. The disadvantage of DESI is its low spatial resolution (a few tens of micrometers). Eberlin et al. [183] showed that MUFA-PC is increased in HCC by using LC–MS and MALDI–MSI, and reported its diagnostic, prognostic, and therapeutic potential. Using DESI–MSI, Banerjee et al. [185] reported that cholesterol sulfate is increased in prostate cancer and its precancerous lesion, and can be used as a biomarker. Thus, MSI can be used to explore the visualization and distribution of individual lipid species, allowing us to investigate various biological processes and dynamic spatial distributions associated with lipid interactions.

## 5. Conclusions

At this time, cancer research has mainly focused on genetic mutations and their expression changes. Recent advances in next-generation sequencers have enabled comprehensive analysis of the genome and transcriptome, which has led to further advances in cancer research. On the other hand, although the importance of the relationship between cancer and lipid metabolism has been recognized, it has been difficult to analyze due to technical problems. However, advances in lipidomics techniques, as described in this review, have allowed us to analyze detailed lipid profiles. It has become clear that the reprogrammed lipid metabolism is not only involved in carcinogenesis through its relationship with oncogenes and tumor suppressor genes but also plays an important role in the interaction with the surrounding cancer environment. Therefore, techniques to analyze the localization of lipids such as MSI may play an important role in elucidating the pathogenesis of cancers. Of the more than 200,000 lipids in the body, only a small fraction can be analyzed at this time [5]. Further analysis of cancer lipid metabolism using more advanced technology is required to develop novel cancer treatments.

## Figures and Tables

**Table 1:** Drugs targeting de novo FA synthesis pathway for cancer treatment.

Target	Drugs	Development Stage	References
ACLY	Hydroxycitric acid	preclinical	[15]
	Cucurbitacin B	preclinical	[16,17]
ACC	TOFA	preclinical	[18,19]
	Soraphen A	preclinical	[20]
	ND-646	preclinical	[21,22]
	MK-4074	clinical phase 1 (for treatment of NAFLD)	[23]
	ND-630	clinical phase 2 (for treatment of NAFLD)	[21,24]
FASN	TVB-2640	clinical phase 2 (monotherapy and/or co-treatment)	[25,26]
	Orlistat	FDA-approved (as an anti-obesity drug)	[27,28]
	C75	preclinical	[29,30]
	GSK2194069	preclinical	[31,32]
	Fasnall	preclinical	[33]
SCD	A939572	preclinical	[34,35]
	MF-438	preclinical	[36,37]
	CAY10566	preclinical	[38,39]
	BZ36	preclinical	[40]
SREBP1	Fatostatin	preclinical	[41]
	FGH10019	preclinical	[42]

NAFLD, nonalcoholic fatty liver disease; SREBP1, sterol regulatory element-binding protein 1.

**Table 2:** Summary of the lipidomics techniques.

Types	Characteristics	Advantages	Limitations	Applications to Cancer Research
Shotgun MS	Infuse sample directly into the MS	Less time-consuming; low cost; high reproducibility	Low sensitivity; incapable of distinguishing isomers	Lung cancer [176]
TLC–MS	Separate samples into individual lipid classes without specialized equipment	Less time-consuming; low cost; high reproducibility	Low separation efficiency; impossible to link TLC and MS	Breast cancer [177]
GC–MS	Suitable for analysis of volatile lipids; commonly used for FA and sterol analysis	High separation efficiency and sensitivity; relatively low cost	Limiting for nonvolatile lipids; requires derivatization	Thyroid cancer [178] Breast cancer [179]
LC–MS	The most commonly used method in lipidomics; able to analyze a wide variety of lipids	High separation efficiency and sensitivity	Organic solvent consumption	HCC [49] Prostate cancer [180] Breast cancer [181]
SFC–MS	Own the properties of both GC and HPLC; possible to select wide range of separation mode	Excellent separation resolution; high throughput; low consumption of organic solvent	Not widely spread	Breast cancer [182]
MALDI–MSI	Ionize sample by coating with matrix and irradiating laser	High resolution; the most established technique	Requires matrix pretreatment	HCC [183] Lung cancer [176]
SIMS–MSI	Ionize sample by irradiating ionized noble gas or metal atoms	High resolution; does not require pretreatment	Difficult to detect intact lipids due to hard ionization	Breast cancer [184]
DESI–MSI	Ionize sample by spraying charged micro droplet	Soft ionization; does not require pretreatment; FFA and lipid mediator detectable	Relatively low resolution	Prostate cancer [185]

DESI, desorption electrospray ionization; FFA, free fatty acid; HPLC, high performance liquid chromatography; MALDI, matrix assisted laser desorption/ionization; MSI, mass spectrometry imaging; SIMS, secondary ion mass spectrometry.
